# TTRAP Is a Novel Component of the Non-Canonical TRAF6-TAK1 TGF-β Signaling Pathway

**DOI:** 10.1371/journal.pone.0025548

**Published:** 2011-09-27

**Authors:** György Várady, Balázs Sarkadi, Károly Fátyol

**Affiliations:** Membrane Research Group, Hungarian Academy of Sciences, Budapest, Hungary; Wayne State University, United States of America

## Abstract

Transforming growth factor-β (TGF-β) principally relays its effects through the Smad pathway however, accumulating evidence indicate that alternative signaling routes are also employed by this pleiotropic cytokine. For instance recently, we have demonstrated that ligand occupied TGF-β receptors can directly trigger the TRAF6-TAK1 signaling module, resulting in MAP kinase activation. Here we report identification of the adaptor molecule TTRAP as a novel component of this non-canonical TGF-β pathway. We show that the protein associates with TGF-β receptors and components of the TRAF6-TAK1 signaling module, resulting in differential regulation of TGF-β activated p38 and NF-κB responses. Modulation of cellular TTRAP level affects cell viability in the presence of TGF-β, suggesting that the protein is an important component of the TGF-β induced apoptotic process.

## Introduction

TGF-β has pervasive and diverse effects on cell physiology as well as it acts as a potent anticancer agent that prohibits uncontrolled cell proliferation [1]–[3]. The most accepted model for the signaling mechanism of TGF-β family cytokines portrays a relatively simple pathway, in which ligand binding to a membrane bound receptor complex induces a conformational change, resulting in phosphorylation and activation of the type I receptor (TβRI) by the type II receptor kinase (TβRII). Through its own kinase activity, TβRI then phosphorylates the appropriate receptor Smads (R-Smads, Smad2/3). Once phosphorylated, R-Smads can form complexes with the common Smad (Smad4), whereupon they translocate to the nucleus to initiate specific transcriptional programs [4], [5]. It is becoming increasingly apparent however, that the picture depicted above is significantly more complex. TGF-β can mobilize several intracellular signal transducers in Smad-independent manner as well [6]–[8]. These non-canonical, non-Smad pathways are also activated directly by ligand-occupied receptors to reinforce, attenuate or otherwise modulate downstream cellular responses. The non-Smad pathways include various branches of MAP kinase pathways, Rho-like GTPase signaling pathways, the phosphatidylinositol-3-kinase/AKT pathway and more. Such alternative signal transducers often regulate the Smad pathway itself and represent extensive opportunities for crosstalk with other signaling routes, contributing to the surprising diversity of TGF-β responses.

Perhaps one of the most important non-Smad pathways is the p38/JNK MAP kinase cascade [9]–[12]. This signaling route functions in conjunction with the Smad pathway to regulate such cellular responses as apoptosis and eptithelial-to-mesenchymal transition (EMT). Despite their obvious biological significance however, we still have serious caveats in understanding the mechanisms by which TGF-β governs them. The need to fill out these gaps is further underscored by several recent observations, suggesting that imbalances arising between the Smad-pathway and the p38/JNK MAPK signaling branches during tumorigenesis may contribute to the conversion of TGF-β from a suppressor to a promoter of cancer growth [13]–[19].

Previous genetic studies placed TGF-β-activated kinase 1 (TAK1) in the TGF-β mediated p38/JNK activation pathway however, the link between TAK1 and the activated receptor complex had been lacking [20]–[22]. Recently, we and others have demonstrated that the E3 ubiquitin ligase, TRAF6 is one of the missing pieces [23], [24]. The molecule physically interacts with the TGF-β receptor complex and is required for Smad-independent activation of the JNK and p38 kinases. TGF-β promotes association between TRAF6 and TAK1, resulting in lysine 63-linked (K63) ubiquitylation and subsequent activation of TAK1. Interestingly, the TRAF6-TAK1 signaling module is also employed by a number of different signaling routes such as those emanating from the IL-1β receptor, Toll-like receptors, T-cell receptor etc. and cellular processes triggered by DNA damage and osmotic stress [25], [26]. Selective activation of TAK1 by the numerous divergent stimuli is believed to be achieved at least in part by the use of adaptor proteins indigenous to a given signaling route and/or employment of unique combinations of more common ones. Regardless, the identification of these adaptor proteins and the elucidation of their complex interactions are essential.

Here we describe one such adaptor molecule, TTRAP (TRAF and TNF receptor associated protein) [27] that may contribute to the specific activation of TAK1 in response to TGF-β. TTRAP was originally reported to interact with members of the TNF receptor family and TRAF adaptor proteins [27]. Subsequent studies also implicated the molecule in various nuclear functions, including transcription and DNA repair [28]–[31]. Notwithstanding, a recent work convincingly demonstrated a role for TTRAP in signal transduction [32]. An antisense screen in zebrafish indentified the protein as a component of the Nodal/activin signaling pathway and an important regulator of embryogenesis. Here we show that TTRAP is involved in TGF-β signaling in mammalian cells as well. Specifically, the protein associates with components of the TGF-β receptor-TRAF6-TAK1 signaling module and promotes their ubiquitylation dependent complex formation. We also demonstrate that TTRAP, by modulating the activities of non-canonical TGF-β induced signaling routes, plays an important role in TGF-β elicited apoptosis.

## Materials and Methods

### Cell culture, transfection, reporter assays

HEK293T, Phoenix-E, NMuMG and AML12 cells were purchased from the American Type Culture Collection and maintained as recommended by the supplier. Cells were transfected with Fugene 6 (Roche) or FugeneHD (Promega), according to the manufacturers instructions. Reporter assays were performed as described earlier [33].

### Antibodies, shRNAs, chemicals

The following antibodies were used in this study: phospho-Smad2(Ser465/467), phospho-p38(Thr180,Tyr182)(D3F9), p38 and phospho-TAK1(Thr187) were from Cell Signaling Technology; Smad2/3(C8), TRAF6(D-10), TAK1(M-579), TTRAP/EAPII(K-13), TTRAP/EAPII(N-18) were from Santa Cruz; Myc(9E10) and HA(3F10) were purchased from Roche; His and FLAG(M2) were from Sigma. Mission shRNA lentiviruses, targeting the mouse TTRAP mRNA (TRCN0000174689, TRCN0000174799 and TRCN0000174910) were purchased from Sigma. Recombinant human TGF-β1 was from R&D Systems. SB431542, SB203582 and SP600125 were obtained from Sigma. The TAK1 inhibitor, (5Z)-7-oxozeaenol was from Calbiochem.

### Plasmids

Most of the expression plasmids used here were described earlier [23]. Full length TTRAP, TAK1 and TAB2 cDNAs were generated by PCR and cloned into the pRK family of mammalian expression vectors [34] using standard techniques. Retroviral expression constructs were created in the pBabe-Puro backbone [35]. Deletion and point mutants were generated by PCR. Sequences of all constructs were verified by sequencing.

### Immunoprecipitation, western blotting

Western blotting of proteins and immunoprecipitations (IP) were performed as described earlier [33].

### Cell viability measurements

Cell viability was assessed by three different methods: **1.** Propidium iodide (PI) uptake of cells, as a measure of membrane integrity, was determined by fluorescence activated cell sorting (FACS). Cells were seeded at a density of 3×10^4^ cells/well in 24-well plates and treated as indicated. Subsequently, cells were collected by trypsinization, washed with BSA-PBS (PBS containing 0.5% BSA) and resuspended in BSA-PBS containing 2 µg/ml PI. The cell suspension was incubated at room temperature for 10 minutes and then measured by FACS. FACS profiles were analyzed by the WinMDI software. **2.** Apoptosis was followed by staining of cells with Alexa Fluor 647 labeled annexin V (Invitrogene) according to the manufacturer's instructions and analyzed by FACS. **3.** Cell survival was also determined by the MTT (3-[4,5-dimethylthiazol-2-yl]-2,5-diphenyltetrazolium bromide) assay. Cells were seeded at a density of 5×10^3^ cells/well in 96-well plates. The following day treatments were commenced as indicated. At the end of the treatments the medium was replaced with fresh medium containing 1.2 mM MTT and the cells were incubated at 37°C in 5% CO_2_ for 4 hours. Subsequently, the cells were washed in the plates with PBS and the formazan crystals were solubilized in isopropanol, containing 0.1 M HCL. Optical densities at 570 nm were measured in a plate reader.

### Indirect immunofluorescence

For indirect immunofluorescence, cells were grown on coverslips and fixed in cold methanol for 7 minutes and then briefly permeabilized in cold acetone. The antibody incubations and washing steps were done as described [33].

## Results

### TTRAP associates with the TGF-β receptor complex

Based on earlier results implicating TTRAP in the signaling processes of Nodal/activin ligands, we explored the protein's potential involvement in TGF-β signaling in mammalian cells. Zebrafish TTRAP has been shown to bind components of the Nodal/activin pathway (the type I Nodal/activin receptor [Alk4], and Smad3). Thus, as an initial approach, we tested the association of mammalian TTRAP with elements of the TGF-β signaling machinery, using various protein-protein interaction techniques.

**Figure 1:**
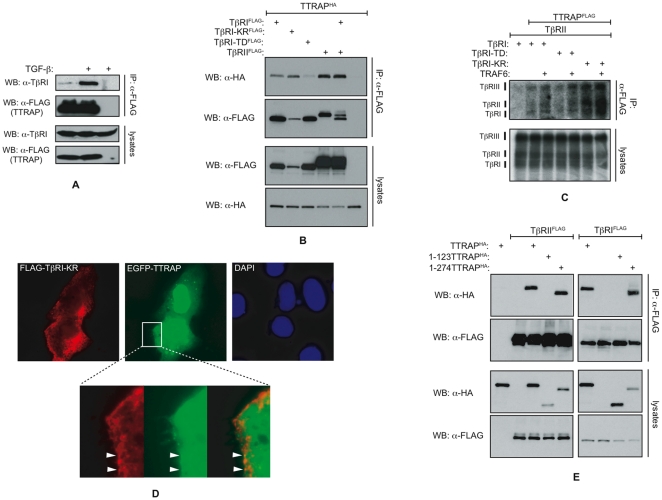
TTRAP interacts with TGF-β receptors. A) TTRAP associates with endogenous TβRI. NMuMG cells stably expressing FLAG-TTRAP were treated with 4 ng/ml of TGF-β for one hour or left untreated. Cellular lysates were prepared and TTRAP was precipitated with FLAG affinity beads. The precipitated proteins and 1/30th of the input lysates were analyzed by western blotting. B) Co-IP analysis of the TTRAP-TGF-β receptor interaction. The indicated proteins were co-expressed in HEK293T cells. Total cellular lysates were prepared and the TGF-β receptors were precipitated with a FLAG antibody. The precipitated proteins and 1/20th of the input lysates were analyzed by western blotting. C) Analysis of the binding of TTRAP to membrane associated TGF-β receptors. To label surface receptors cells were incubate with [^125^I]-TGF-β, cross-linked with DSS and TTRAP was pulled down. The precipitated receptors were detected by autoradiography. D) EGFP-TTRAP and FLAG-TβRI-KR were co-expressed in AML12 cells and their localizations were monitored by fluorescence microscopy. A juxta-membrane region of the cell was zoomed out at the bottom. Co-localized foci are indicated by arrowheads. The nuclei were stained by 4′,6-diamidino-2-phenylindole (DAPI). E) Mapping of the TGF-β receptor binding domain of TTRAP by co-IP. The precipitated proteins and 1/20th of the input lysates were analyzed by western blotting using HA and FLAG antibodies.

First, we wanted to analyze the interaction between endogenous TTRAP and the TGF-β receptor complex. Unfortunately, currently there is no commercially available TTRAP antibody sensitive enough to carry out such studies. To circumvent this problem, we generated an NMuMG cell population stably expressing FLAG epitope tagged TTRAP (FLAG-TTRAP) at a modest level. Using these cells, we were able to detect modest binding of FLAG-TTRAP to endogenous TβRI by co-immunoprecipitation (co-IP) (Figure 1A). Importantly, this interaction was significantly increased upon TGF-β treatment.

Second, epitope tagged TTRAP and TGF-β receptors were transiently co-expressed in HEK293T cells and their interactions were analyzed by co-IP (Figure 1B). Under these conditions, TTRAP associated with both TβRI and TβRII even in the absence of TGF-β stimulation. The protein exhibited increased affinity toward the catalytically inactive TβRI-KR receptor mutant compared to the constitutively active TβRI-TD form.

Third, the binding of TTRAP to TGF-β receptors was monitored *in vitro* using GST pull-down. HA-tagged TTRAP protein was synthesized in rabbit reticulocyte lysate *in vitro*, while GST-tagged cytoplasmic domains of TβRI and TβRII were produced in bacteria. *In vitro* TTRAP bound to the cytoplasmic domains of both TGF-β receptors immobilized on gluthatione beads, indicating that their interactions are direct (Figure S1).

Fourth, we evaluated the binding of TTRAP with membrane associated TGF-β receptor complexes. HEK293T cells were co-transfected with TTRAP, TGF-β receptors and TRAF6 in various combinations. Subsequently, surface proteins were affinity labeled with [^125^I]-TGF-β. Following cross-linking with disuccinimidyl suberate (DSS) the cells were lysed and TTRAP was precipitated with a FLAG antibody. As shown in Figure 1C, TTRAP pulled down [^125^I]-TGF-β occupied TGF-β receptor complexes. Importantly, the relative binding affinities of TTRAP toward the various mutant forms of TβRI detected by this technique were similar to those seen in co-IPs. In addition, we noted that the presence of TRAF6 strengthened the interaction between TTRAP and the TGF-β receptor complex (see also later).

Fifth, EGFP-TTRAP and FLAG-TβRI-KR were co-expressed in AML12 cells and their localizations were monitored by fluorescence microscopy. TTRAP was present both in the cytoplasm and the nucleus, consistent with previous reports [28], [29]. Significantly, a fraction of the cytoplasmic TTRAP exhibited co-localization with TβRI in juxta-membrane foci (Figure 1D).

Finally, the TGF-β receptor interacting domain of TTRAP was mapped by co-IPs. Using C-terminally truncated TTRAP molecules we showed that the region between amino acids 123 and 274 is necessary for TGF-β receptor binding (Figure 1E). Interestingly, this region of TTRAP is part of the evolutionary conserved exo/endo/phos domain also present in a number of Mg^2+^/Mn^2+^ dependent phosphodiesterases [36].

In summary, the above results indicate that in analogy to the TTRAP-Alk4 interaction observed in zebrafish, the mammalian ortholog of TTRAP associates with TGF-β receptors. The fact that TTRAP also binds with ligand occupied TGF-β receptor complexes on the cell surface provides further support for the physiological relevance of these interactions. Contrary to previous data however, we were unable to detect direct binding of TTRAP with Smads (Figure S2).

### TTRAP associates with the TAK1 complex

**Figure 2:**
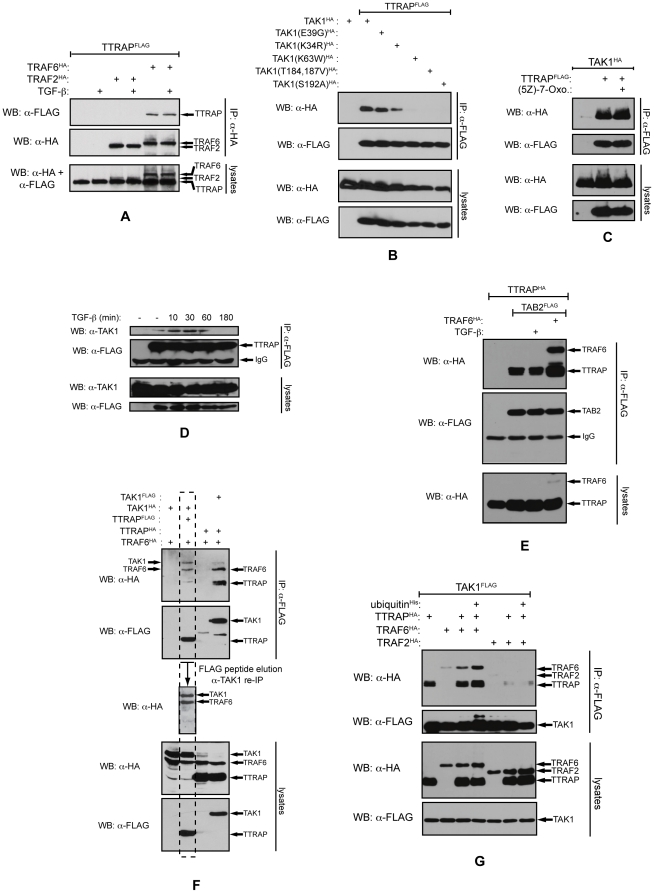
TTRAP associates with the TAK1 complex. A) TTRAP associates with TRAF6. The indicated proteins were expressed in HEK293T cells. Total cellular lysates were prepared and TRAFs were pulled down. The precipitated complexes were analyzed by western blotting. B) TTRAP binds to TAK1. The indicated proteins were co-expressed in HEK293T cells. TTRAP was precipitated from the cellular lysates and the co-precipitation of TAK1 was analyzed by western blotting. C) TAK1 kinase activity is not required for TTRAP binding. Transfected HEK293T cells were treated with 0.5 µM (5Z)-7-oxozeaenol. TTRAP was precipitated from the lysates and the co-precipitating TAK1 molecules were detected. D) TTRAP associates with endogenous TAK1. An NMuMG cell population was established stably expressing FLAG-TTRAP. FLAG-TTRAP was precipitated from the TGF-β treated cells and the co-purifying endogenous TAK1 was detected by western blotting. E) TTRAP interacts with TAB2. TAB2 was precipitated from transfected HEK293T cells and the protein complexes were analyzed by western blotting. F) Ternary complex formation of TTRAP, TAK1 and TRAF6. TAK1 or TTRAP was precipitated from transfected HEK293T cells with a FLAG antibody and the co-precipitation of the other two molecules were analyzed by an HA antibody. In the co-IP - indicated by a dashed box - the TTRAP complexes were eluted from the agarose beads by a large excess of FLAG peptide and subjected to a second round of IP with a TAK1 antibody. Co-precipitation of TAK1 and TRAF6 was monitored by western blotting. G) TRAF2 can not substitute for TRAF6 in the TAK1-TTRAP-TRAF6 complex. FLAG-TAK1 containing complexes were pulled down from transfected HEK293T cells. The precipitated proteins were analyzed by western blotting.

TTRAP was originally identified as a TRAF interacting protein. Amongst members of the TRAF family, it exhibited the highest affinity toward TRAF6 and practically no binding with TRAF2 [27]. Indeed, using co-IP, we were able to verify these observations (Figure 2A). Given that TRAF6 plays a crucial role in TGF-β induced p38 activation, next TTRAP's interactions with other components of the TGF-β receptor-p38 pathway were examined. First, we tested whether TTRAP can interact with TAK1. Co-IP was employed to assess the associations between FLAG-TTRAP and HA epitope tagged TAK1 molecules (wild-type and various mutant forms) (Figure 2B). TTRAP bound avidly to catalytically active TAK1 variants (both the wild-type and the E39G point mutant) however, replacement of lysine-34 - the major acceptor site for TGF-β induced K63-linked polyubiquitylation [24] - with an arginine residue, significantly reduced this interaction. The affinity of TTRAP toward catalytically inactive mutants of the kinase was diminished even further, exhibiting no significant binding to either the ATP binding site mutant (K63W) or the activation loop mutants (T184,187V and S192A) [37], [38]. This finding raised the possibility that TTRAP specifically binds to auto-phosphorylated residues in the kinase. Specific inhibition of TAK1's catalytic activity with (5Z)-7-oxozeaenol [39] however, did not abolish the TAK1-TTRAP association (Figure 2C). This suggests that TTRAP recognizes some structural feature of the kinase associated with its catalytically active form, rather than the phosphorylated residues per se.

To analyze the interaction between TTRAP and TAK1 under more physiological settings, we employed the NMuMG cell population stably expressing FLAG-TTRAP mentioned earlier. In these cells, we were able to detect a dynamic interaction between endogenous TAK1 and FLAG-TTRAP (Figure 2D). The weak basal TTRAP-TAK1 association was enhanced by TGF-β treatment, peaked at ∼30 minutes and was almost completely diminished by 180 minutes.

In the cells the activity of TAK1 is strictly regulated by various TAK1 binding proteins (TABs) [40]–[43]. Importantly, some of these TABs have also been implicated in TGF-β signaling. Thus, the interactions of TTRAP with two such TABs, TAB1 and TAB2 were tested by co-IP. We found that TTRAP did not bind to TAB1 (data not shown). Conversely, the protein showed strong interaction with TAB2, which was enhanced even further by the co-expression TRAF6 (Figure 2E).

Next, the TAK1 binding domain of TTRAP was mapped by co-IP. We showed that the N-terminal 1–123 aa segment of TTRAP was sufficient for this interaction (Figure S3A). Given that TTRAP is using a distinct region to bind TRAF6 (124–274 aa, Figure S3B), it is possible that the protein can interact with TAK1 and TRAF6 simultaneously in a ternary complex. Indeed, pulling down either protein (TTRAP, TAK1, or TRAF6) co-precipitated the other two in approximately equal quantities (Figure 2F). To provide further support for the existence of the TAK1-TTRAP-TRAF6 ternary complex, sequential co-IPs were performed (Figure 2F). FLAG-TTRAP was co-expressed with HA-TAK1 and HA-TRAF6 in HEK293T cells. After 36 hours, cell lysates were prepared and TTRAP complexes were purified on FLAG affinity beads. An aliquot of the precipitated material was used for western analysis to confirm that both TAK1 and TRAF6 were co-purified with TTRAP. From the remaining sample TTRAP complexes were eluted with a large excess of FLAG-peptide and used for a second round of IP with a TAK1 antibody. Western analysis demonstrated that TRAF6 efficiently co-precipitated with TAK1 from this eluate, strongly suggesting that TAK1, TTRAP and TRAF6 are capable of forming stable ternary complexes in the cell.

Members of the TRAF adaptor protein family display significant similarity to each other and are all involved in cellular signaling [44]. It has been reported that in some signaling pathways they may also share similar functions and act redundantly. For instance, in the CD40 pathway TRAF2 and TRAF6 are closely collaborating with each other and perform partially overlapping tasks [45]. Therefore, the ability of TRAF2 to substitute for TRAF6 in the protein complexes described above was also examined. As seen in Figure 2G TRAF2, unlike TRAF6, did not display significant affinity toward TAK1. Conversely, TRAF2 was even capable of disrupting the TAK1-TTRAP interaction, emphasizing the specific role TRAF6 plays in the above complexes.

### TTRAP is ubiquitylated by TRAF6 and promotes TRAF6 dependent ubiquitylation of TAK1

**Figure 3:**
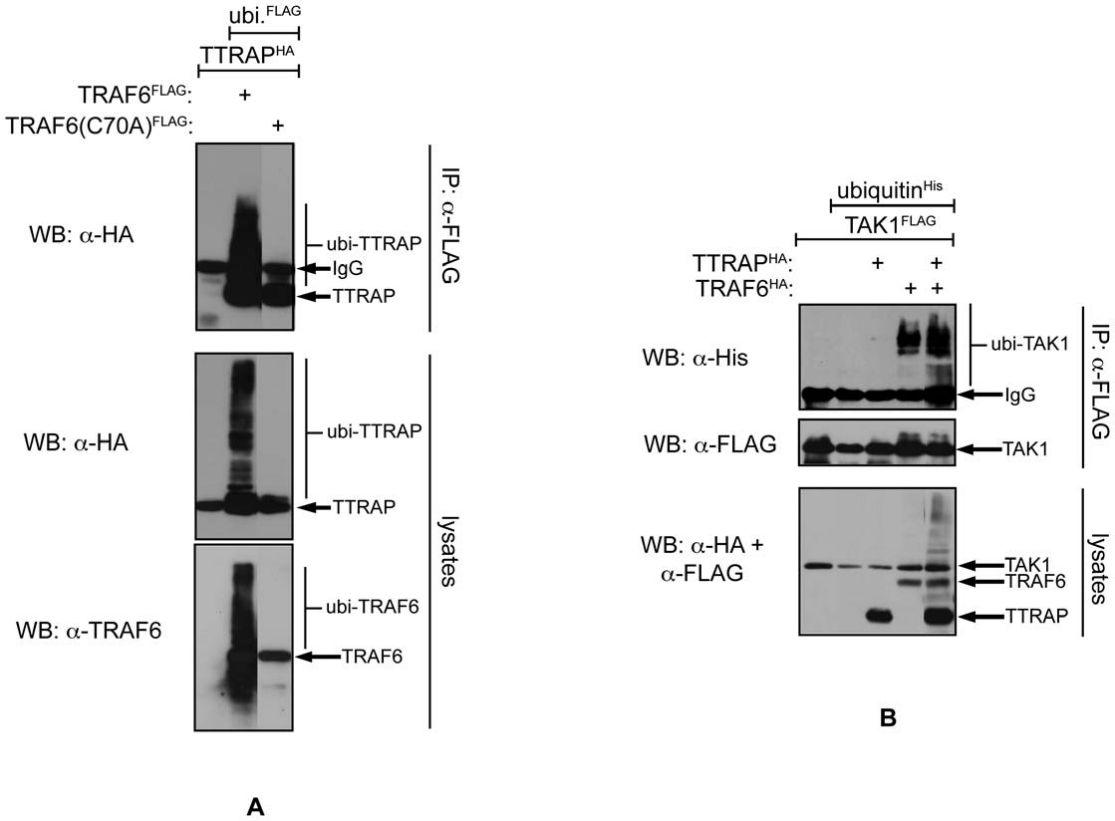
TTRAP is ubiquitylated by TRAF6 and promotes TRAF6 dependent ubiquitylation of TAK1. A) FLAG tagged proteins were pulled down from transfected HEK293T cells and the precipitating TTRAP protein was detected by western blotting using an HA antibody. B) Tranfected HEK293T cells were lysed in 0.5% hot SDS. The lysates were diluted with IP buffer and TAK1 was pulled down. Ubiquitylated TAK1 was detected by western blotting using a His-tag antibody. The input lysates were also analyzed by western blotting using the indicated antibodies.

TRAF6 is an E3 ubiqutin ligase capable of catalyzing the formation of K63-linked polyubiquitin chains [46]. To test whether TRAF6 can ubiquitylate TTRAP an *in vivo* ubiquitylation assay was performed (Figures 3A and S4). HA-TTRAP was co-expressed with FLAG-ubiquitin and various forms of TRAF6 in HEK293T cells. After 36 hours, the cells were lysed and the ubiquitylated proteins - purified from the lysates on FLAG affinity beads - were subjected to western blot analysis. High molecular weight HA antibody reactive bands, corresponding to polyubiquitylated TTRAP molecules, were only detected when wild-type TRAF6 was co-expressed in the cells. The RING domain mutant TRAF6(C70A) failed to promote the ubiquitylation of TTRAP, consistent with its inability to catalyze its own ubiquitylation.

**Figure 4:**
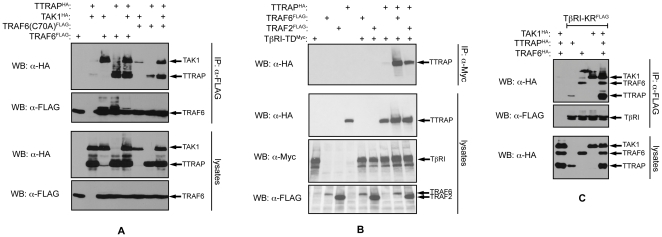
The TAK1-TTRAP-TRAF6 complex is stabilized by ubiquitylation and recruited to TβRI. A) FLAG-TRAF6 was precipitated from transfected HEK293T cells and the co-precipitation of TAK1 and TTRAP was examined by western blotting. B, C) The indicated epitope tagged proteins were co-expressed in HEK293T cells. TβRI was pulled down from the lysates and the co-precipitating TTRAP, TRAF6 and TAK1 were analyzed by western blotting.

We noted that co-expression of TTRAP with TRAF6 and TAK1 increases the amount of high molecular weight TAK1 forms, most likely representing ubiquitylated molecules (see for example Figure 4). Thus, we tested the possibility whether TTRAP can enhance TRAF6 mediated TAK1 ubiquitylation. FLAG-TAK1 was co-expressed with HA-TRAF6, HA-TTRAP and His-ubiquitin in cells in various combinations. To disrupt non-covalent protein complexes cellular lysates were prepared in hot 0.5% SDS solution. FLAG-TAK1 was purified from the diluted lysates on FLAG affinity beads and ubiquitylated TAK1 was detected in western blot using a His-tag antibody. As seen in Figure 3B, co-expression of TTRAP indeed increased the E3 ubiquitin ligase activity of TRAF6 toward TAK1.

### The TAK1-TTRAP-TRAF6 complex is stabilized by ubiquitylation and recruited to TβRI

TRAF6 has been shown to promote the formation of signaling complexes, by at least partly depending on its E3 ubiquitin ligase activity [46]–[48]. Since TRAF6 ubiquitylates both TTRAP and TAK1, we examined the possible role of this modification in the stabilization of the TAK1-TTRAP-TRAF6 ternary complex. To this end, complex forming ability of the wild type TRAF6 protein was compared with that of the catalytically deficient C70A RING domain mutant (Figure 4A). In co-IPs wild type TRAF6 efficiently pulled down both TAK1 and TTRAP. Importantly, in these samples high molecular weight forms of each protein - corresponding to ubiquitin modified molecules - could also easily be detected. In contrast, the interactions of TRAF6(C70A) with both TAK1 and TTRAP were strongly diminished. Concurrently, ubiquitylated molecules were also mostly missing from the latter samples. Co-expression of TAK1 and TTRAP synergistically increased each other's affinity toward TRAF6(C70A) however, even in this case mutant TRAF6 interacted less efficiently with the two proteins than the wild type, suggesting that ubiquitin mediated interactions also contribute to the stabilization of the TAK1-TTRAP-TRAF6 complex.

Ligand engagement of numerous cytokine receptors leads to the assembly of multiprotein signaling complexes on their intracellular domains. Members of the TRAF adaptor protein family have been shown to play crucial role in these processes [44]. Prompted by these observations, we tested whether TRAF6 can influence TTRAP's association with the TGF-β receptors using co-IPs (Figure 4B). As described above, TTRAP exhibited relatively weak binding to TβRI-TD. Co-expression of TRAF6 however, dramatically increased TTRAP's affinity toward the receptor. Importantly, the increased binding was accompanied by the appearance of ubiquitylated TTRAP forms, suggesting that TRAF6 mediated ubiquitylation may contribute to the stabilization of the TTRAP-TβRI complex. Similarly to TRAF6, ectopic expression of TRAF2 also increased the TTRAP-TβRI association, though to a much-reduced degree, indicating that under physiological conditions TRAF2's role may be negligible in the stabilization of the TTRAP-TGF-β receptor complex.

Binding of TAK1 with TGF-β receptors has been demonstrated by several studies [23], [24], [49], [50]. We examined how this interaction is influenced by TTRAP and TRAF6. As shown in Figure 4C, ectopic expression of TRAF6 helped the recruitment of not only TTRAP but TAK1 as well to TβRI. Notably, enrichment of ubiquitylated forms of the proteins could also be observed in the TβRI immunoprecipitates, indicating that the complexes might be stabilized by this modification.

### TTRAP is involved in non-canonical TGF-β signaling

**Figure 5:**
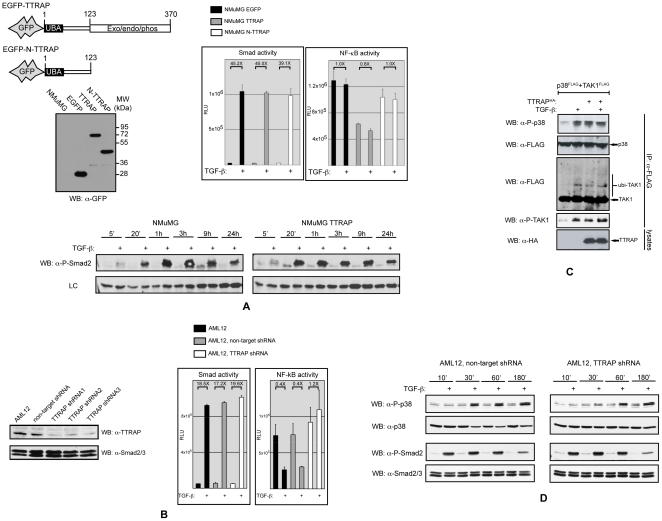
TTRAP is involved in non-canonical TGF-β signaling. A) Stable NMuMG cell populations expressing GFP, GFP-tagged full-length TTRAP (TTRAP) and GFP-tagged 1–123 aa TTRAP (N-TTRAP) proteins were generated by retroviral transduction. Transduced, GFP positive cells were sorted by FACS. Expressions of the introduced genes were verified by western blotting (top left). The above NMuMG cell lines were transfected with Smad (3TP-lux) and NF-κB reporters (top right). The cells were treated with 4 ng/ml of TGF-β for 16 hours and firefly luciferase activites were measured. To take into account the different transfection efficiencies, a Renilla luciferase expressing plasmid was co-transfected with the reporters. Subsequently, the firefly luciferase activities were normalized for Renilla luciferase activities. The error bars represent standard deviations. Smad2 phosphorylation was monitored in the parental and TTRAP expressing NMuMG cells by western blotting (bottom). A non-specific band is shown as a loading control. B) Endogenous TTRAP level was reduced in AML12 cells by transduction of lentiviruses expressing shRNAs specific for the murine TTRAP mRNA. Transduced, GFP positive cells were enriched by FACS. Lentiviruses expressing three different TTRAP shRNAs were used to rule out off-target effects. A virus vector expressing a non-target shRNA was also employed as a control. In the transduced cell populations TTRAP level was monitored by western blotting (left). Smad and NF-κB transcriptional activities were measured as above in parental and shRNA expressing AML12 cells (right). C) Transfected HEK293T cells were treated with 4 ng/ml of TGF-β for 30 minutes before cell lysis. TAK1 and p38 were precipitated from the lysates and their phosphorylation status was monitored by western blotting. D) Smad2 and p38 phosphorylation were examined in non-target and TTRAP shRNA expressing AML12 cells. Comparable sample loading was also monitored using p38 and Smad2/3 antibodies.

Having established that TTRAP interacts with TGF-β receptors and components of the TRAF6-TAK1 signaling module, we wanted to evaluate the protein's involvement in various TGF-β induced biological responses. We started with the establishment of stable NMuMG cell populations expressing the EGFP-tagged full-length TTRAP molecule (TTRAP cells). As controls, cells were also produced expressing the N-terminal 123 aa fragment of TTRAP tagged with EGFP (N-TTRAP cells) or EGFP alone (EGFP cells) (Figure 5A). Smad-dependent transcription was monitored in the above cells using the 3TP-lux reporter. TGF-β treatment resulted in the same degree of Smad activation in all cell lines and consistently, TβRI mediated Smad2 phosphorylation also followed a similar kinetics (Figure 5A). Earlier studies suggested that TTRAP is a negative modulator of NF-κB [27]. Thus, we examined the protein's influence on TGF-β induced NF-κB activation as well. TGF-β treatment did not have a significant effect on the activity of an NF-κB reporter in NMuMG cells. However, in TTRAP over-expressing cells the basal NF-κB activity was approximately half of that observed in the control EGFP or N-TTRAP expressing cells (Figure 5A).

The effect of TTRAP deficiency on TGF-β induced transcriptional responses was also examined. These studies were performed in AML12 normal murine hepatocytes, in which the endogenous TTRAP protein level was knocked down by lentiviruses expressing shRNAs specific for the mouse TTRAP gene (Figure 5B). Down-regulation of TTRAP did not have an effect on TGF-β induced Smad-dependent transcription and the kinetics of Smad2 phosphorylation was not affected either (Figure 5B and D). In the parental and the non-target shRNA expressing AML12 cells, TGF-β treatment significantly reduced the activity of an NF-κB reporter in keeping with an earlier report [51]. TTRAP deficiency completely abolished this inhibitory effect and even a slight increase in the basal NF-κB activity could be seen in the TTRAP shRNA expressing cells (Figure 5B).

TTRAP associates with components of the TRAF6-TAK1 signaling module, which plays an essential role in TGF-β induced p38 activation. Additionally, the protein has recently been implicated in proteasome impairment elicited activation of p38 and JNK [52]. In light of these observations, we examined TTRAP's role in TGF-β induced activation of these kinases. Ectopic expression of TTRAP in HEK293T cells activated p38 however, it did not affect JNK phosphorylation (Figure 5C and data not shown). Accompanying p38 activation, ubiquitylation and phosphorylation of TAK1 was also observed. In many cell lines TGF-β activates p38 in two waves [53], [54]. The early stage - peeking between 15–45 minutes - is Smad-independent, while the delayed p38 response - reaching its maximum at 1.5–2 hours - requires Smad-dependent transcription. As shown in Figure 5D, shRNA mediated knockdown of TTRAP expression strongly inhibited the early p38 phosphorylation in AML12 cells, while the delayed p38 activation and Smad2 phosphorylation remained unaffected. In summary, the above data strongly suggest that TTRAP is an important component of Smad-independent non-canonical TGF-β induced signaling responses, principally the p38 kinase cascade and the NF-κB pathway.

### TTRAP plays a role in TGF-β induced apoptosis

**Figure 6:**
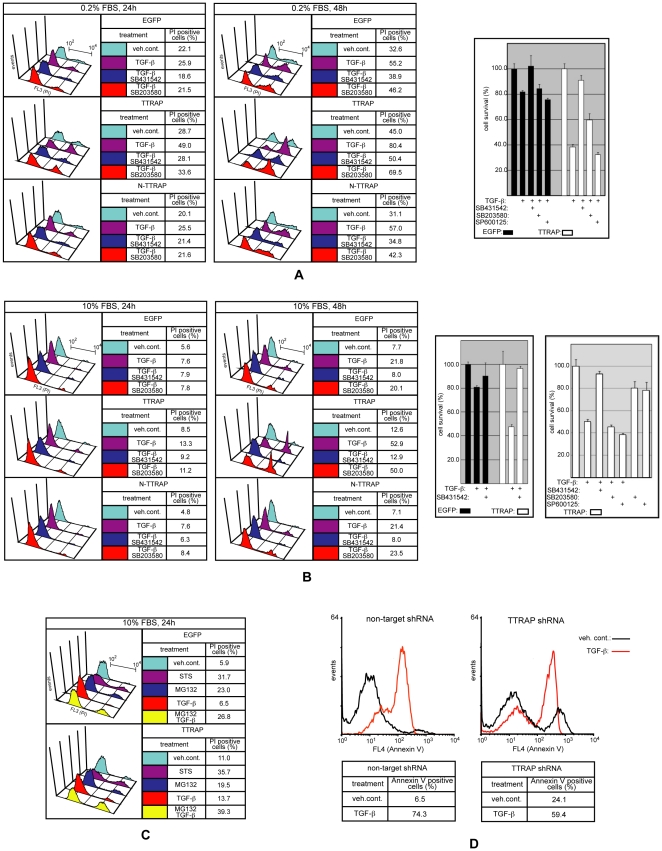
TTRAP is involved in TGF-β induced apoptosis. The stable NMuMG cell populations described in Figure 5 were treated in 0.2% (A) or 10% (B) FBS containing medium as indicated and cell viability was assessed using two different methods. PI uptake of cells, as a measure of membrane integrity, was monitored by FACS (left panels). The experiments were repeated at least twice with similar outcome. On the right side MTT assays were used to measure cell viability. The chemicals used at the following concentrations: TGF-β 4 ng/ml; SB431542, SB203580 and SP600125 were all used at 10 µM. The error bars represent standard deviations. C) NMuMG cells stably expressing TTRAP or EGFP were treated as indicated and integrity of their membranes was monitored by PI uptake. The chemicals used at the following concentrations: TGF-β 4 ng/ml, staurisporine (STS) 1 µM, MG132 2.5 µM. Experiments were repeated several times and a representative result is shown. D) Non-target and TTRAP shRNA lentivirus transduced AML12 cells were treated as indicated in 10% FBS medium. After 24 hours, cells were stained with annexin V and analyzed by FACS.

The NMuMG mammary epithelial cell line has been a well-characterized model system for TGF-β induced apoptosis [11], [23], [55]. TGF-β regulates this process in NMuMG cells through both Smad-dependent and -independent mechanisms, with the Smad-independent component predominantly involving the p38 MAP kinase cascade. To asses the role of TTRAP in TGF-β induced apoptosis, we treated the TTRAP expressing and control NMuMG cells described above with TGF-β under various conditions and subsequently their viability was measured by PI uptake and MTT assay. In accordance with published data, TGF-β elicited only modest apoptosis in the control cell populations (EGFP and N-TTRAP cells) after 24 hours under low-serum (0.2% FBS) culture conditions (Figure 6A) [11], [55]. In contrast, after 24 hours, TTRAP cells exhibited robust TGF-β induced cell death (∼50%), which by 48 hours increased even further (∼80%). By the same time, the TGF-β elicited apoptotic rate in the control cell populations was ∼2/3rd of that of the TTRAP cells. Importantly, TGF-β dependent apoptosis was completely preventable by the TβRI receptor kinase inhibitor, SB431542 and the p38 inhibitor, SB203580 also provided strong protection. The JNK inhibitor, SP600125 did not have a significant effect on the viability of TGF-β treated NMuMG cells. In 10% FBS medium, 24 hours of TGF-β treatment was not able to elicit significant degree of cell death in any of the NMuMG cell lines used, and even after 48 hours only weak apoptosis (∼20%) was detectable in the control cells (Figure 6B). In contrast, under the same conditions in the TTRAP cells the apoptotic rate was >50% by 48 hours and whereas the TβRI inhibitor was still able to prevent apoptosis, the p38 inhibitor lost its protective effect.

Next, we wished to examine the involvement of TTRAP in apoptotic processes induced by other death-promoting stimuli (Figure 6C). TTRAP cells exhibited similar sensitivity to the kinase inhibitor, staurosporine and the proteasome inhibitor, MG132 as the control EGFP cells. Interestingly however, while TGF-β alone was unable to elicit a significant degree of apoptosis after 24 hours under high serum growth conditions in either cell lines, the combined TGF-β/MG132 treatment resulted in synergistic killing of the TTRAP cells.

Finally, TGF-β induced cell death was examined in AML12 hepatocytes made deficient for TTRAP with the use of gene specific shRNAs (see above). In keeping with a recent report [56], decreasing cellular TTRAP level resulted in increased basal apoptosis (∼6% versus ∼24%) (Figure 6D). Importantly however, the robust TGF-β induced cell death was significantly attenuated by TTRAP deficiency (∼74% versus ∼59%), confirming that the protein fulfills a TGF-β dependent pro-apoptotic role in the cells.

## Discussion

In this paper we indentify the TTRAP adaptor molecule as a binding partner for key components of the TRAF6-TAK1 signaling module and the TGF-β receptor complex, strongly suggesting the protein's involvement in non-canonical TGF-β signaling responses. Support for this hypothesis is provided by the following observations: **1.** TTRAP interacts with endogenous TAK1 and TβRI in a TGF-β inducible fashion; **2.** TTRAP forms a stable ternary complex with TAK1 and TRAF6, which can interact with the TGF-β receptor complex; **3.** the E3 ubiquitin ligase activity of TRAF6 is boosted by TTRAP, thereby promoting the ubiquitylation of TAK1 and itself; **4.** ectopic over-expression of TTRAP results in phosphorylation of TAK1 and p38, while it negatively modulates the activity of NF-κB ; **5.** shRNA mediated knockdown of endogenous TTRAP abolishes TGF-β induced rapid phosphorylation of p38 and repression of NF-κB.

TGF-β controls apoptosis through both Smad-dependent and -independent signaling routes. Amongst the Smad-independent pathways, the p38 kinase cascade is generally considered to be a pro-apoptotic pathway, while the NF-κB route has been shown to protect cells from TGF-β induced apoptosis [16], [17], [51], [57]–[59]. Thus, it was not surprising to find that ectopic expression of TTRAP sensitized cells to TGF-β induced cell death, while its down-regulation provided partial protection. The ability of TTRAP to differentially modulate two signaling pathways with opposing outcomes is intriguing however, by no means unique. Recently, the XIAP interacting protein Siva1 was demonstrated to inhibit XIAP and TAK1-TAB1 mediated NF-κB activation, while prolonged TNFα-induced JNK activation resulting in enhanced apoptosis [60]. Interestingly, Siva1 similarly TTRAP, was also shown to interact with the TAK1 complex. Thus, we suggest that TTRAP, along with Siva1, belongs to a group of proteins defined by their abilities to modulate the balance between pro-survival and pro-apoptotic pathways by interacting with the TAK1 complex.

Several works implicated TTRAP in the regulation of apoptosis and depending on the cellular context and the death promoting stimuli used, both pro- and anti-apoptotic properties have been attributed to the protein [58], [56], [61]. Our results not only establish TTRAP as a novel component of the non-canonical TRAF6-TAK1 signaling branch of TGF-β signaling, but also demonstrate its specific involvement in TGF-β induced apoptosis. It is becoming increasingly clear that imbalances arising during tumor progression between various branches of TGF-β signaling conspire to convert TGF-β from a suppressor of tumor formation to a promoter of their growth. Thus, one may hypothesize that restoration of this equilibrium could be of great therapeutic value. From this perspective, the TRAF6-TAK1 signaling module could be a unique and very attractive target for intervention. This module is a point of convergence for both pro-apoptotic (p38/JNK MAP kinase cascades) and pro-survival (NF-κB and PI3K/Akt pathways) signaling routes. Since TTRAP interacts with all key components of this module, thorough understanding of its mode of action may help us formulate strategies for steering the TGF-β pathway in different directions, favoring either survival or apoptosis.

## Supporting Information

Figure S1 ***In vitro*** **interaction of TTRAP with TβRI and TβRII.** Cytoplasmic domains of TGF-β receptors fused with GST were produced in bacteria and affinity purifed on gluthatione beads. HA-TTRAP, FLAG-TRAF2 and -6 were produced by *in vitro* translation in rabbit reticulocyte lysates. *In vitro* binding of TTRAP and TRAFs to gluthatione bead-bound GST, GST-TβRI-CD and GST-TβRII-CD were examined by western blotting.
(TIF)

Figure S2 **Analysis of the binding of TTRAP with Smads by co-IP.** TTRAP was pulled-down from transfected HEK293T cells and the co-precipitation of the Smads and TβRI was examined by western blotting.
(TIF)

Figure S3 **Mapping of the TRAF6 and TAK1 binding domains of TTRAP by co-IP.** A, B) TRAF6 or TAK1 was precipitated from transfected HEK293T cells and the co-precipitating TTRAP fragments were detected by western blotting. Note that TAK1 and EGFP-TTRAP has similar eletrophoretic mobilities, thus the HA reactive band in lane 2 of IP panel A corresponds to a mixture of the two molecules.
(TIF)

Figure S4 **TRAF6 promotes the ubiquitylation of TTRAP.** Original scans for Figure 3A.
(TIF)
