# Supplementary figures and images for: TTRAP Is a Novel Component of the Non-Canonical TRAF6-TAK1 TGF-β Signaling Pathway
Source: PLoS One. 2011 Sep 27;6(9):e25548. doi: 10.1371/journal.pone.0025548 (PMC3182262; doi:10.1371/journal.pone.0025548)

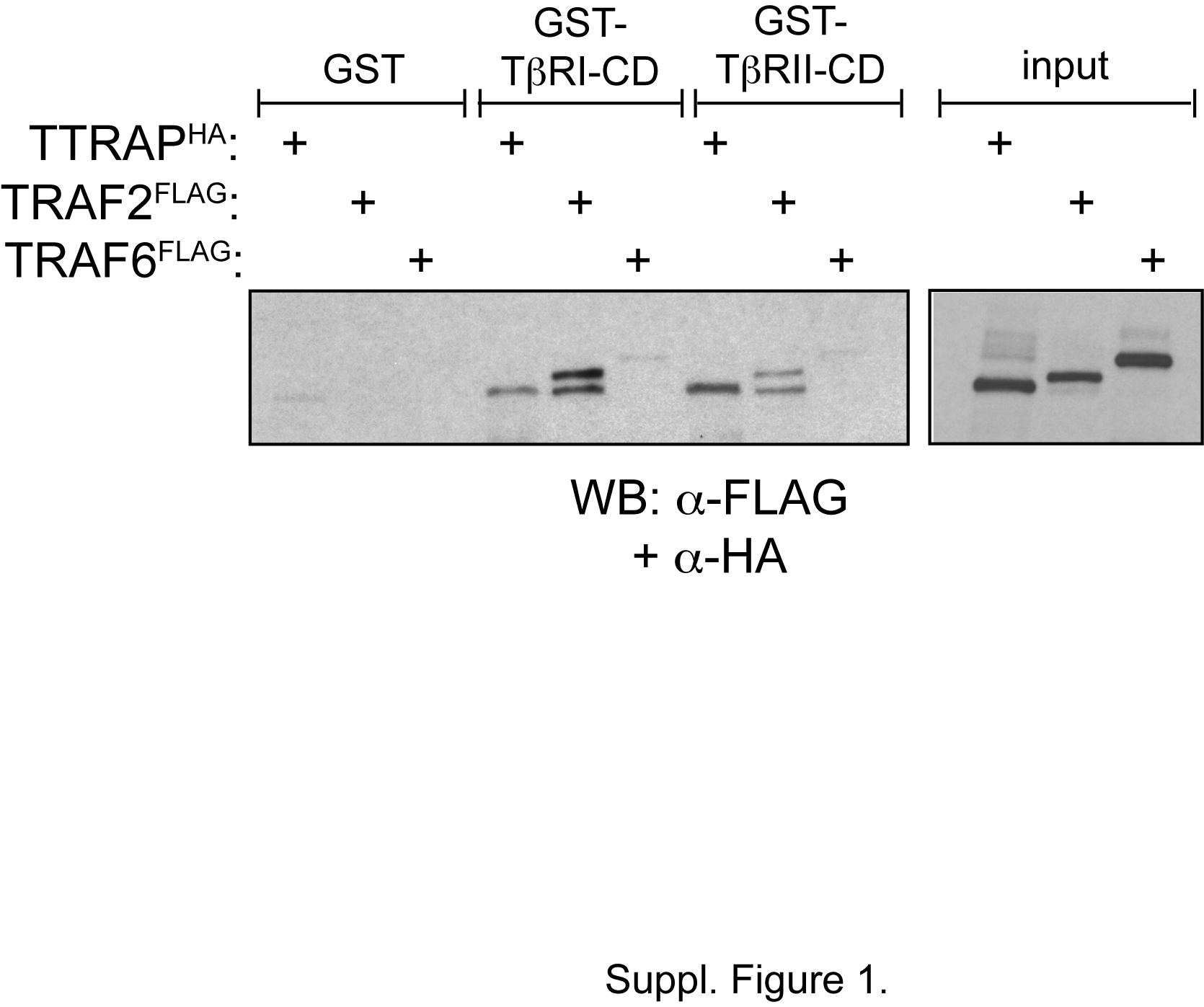

Supplement: Figure S1 — In vitro interaction of TTRAP with TβRI and TβRII. Cytoplasmic domains of TGF-β receptors fused with GST were produced in bacteria and affinity purifed on gluthatione beads. HA-TTRAP, FLAG-TRAF2 and -6 were produced by in vitro translation in rabbit reticulocyte lysates. In vitro binding of TTRAP and TRAFs to gluthatione bead-bound GST, GST-TβRI-CD and GST-TβRII-CD were examined by western blotting. (TIF) [file pone.0025548.s001.tif]

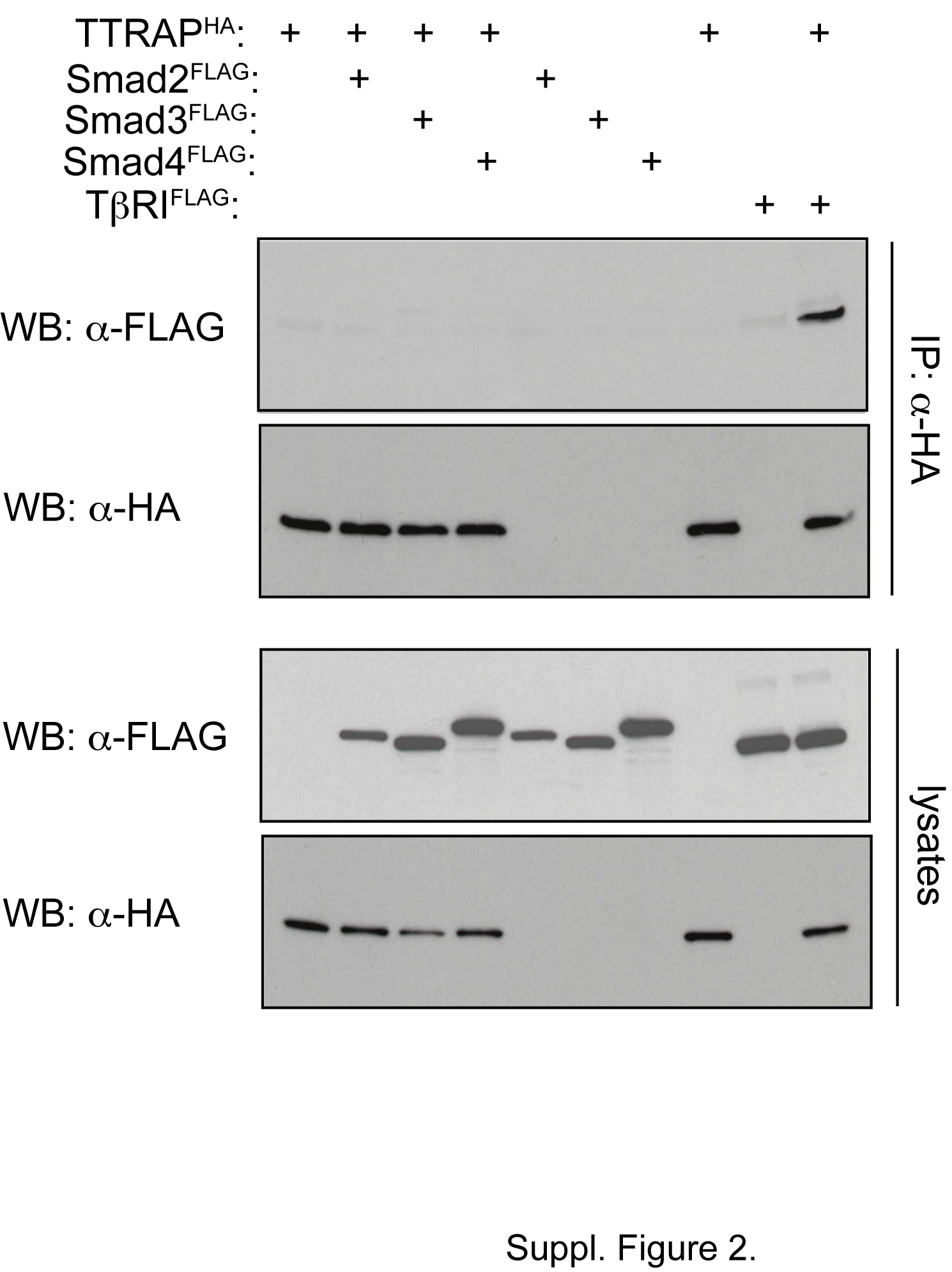

Supplement: Figure S2 — Analysis of the binding of TTRAP with Smads by co-IP. TTRAP was pulled-down from transfected HEK293T cells and the co-precipitation of the Smads and TβRI was examined by western blotting. (TIF) [file pone.0025548.s002.tif]

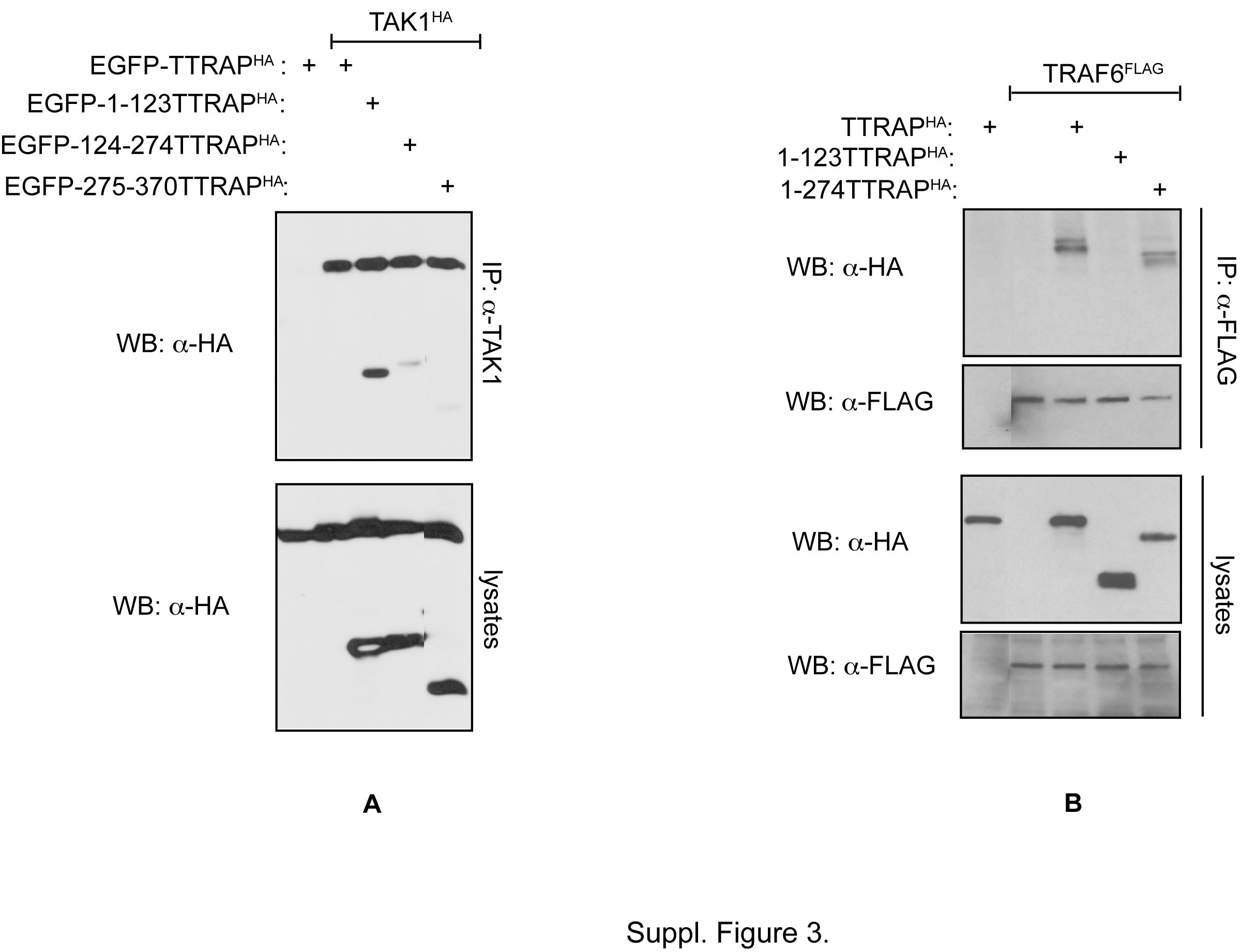

Supplement: Figure S3 — Mapping of the TRAF6 and TAK1 binding domains of TTRAP by co-IP. A, B) TRAF6 or TAK1 was precipitated from transfected HEK293T cells and the co-precipitating TTRAP fragments were detected by western blotting. Note that TAK1 and EGFP-TTRAP has similar eletrophoretic mobilities, thus the HA reactive band in lane 2 of IP panel A corresponds to a mixture of the two molecules. (TIF) [file pone.0025548.s003.tif]

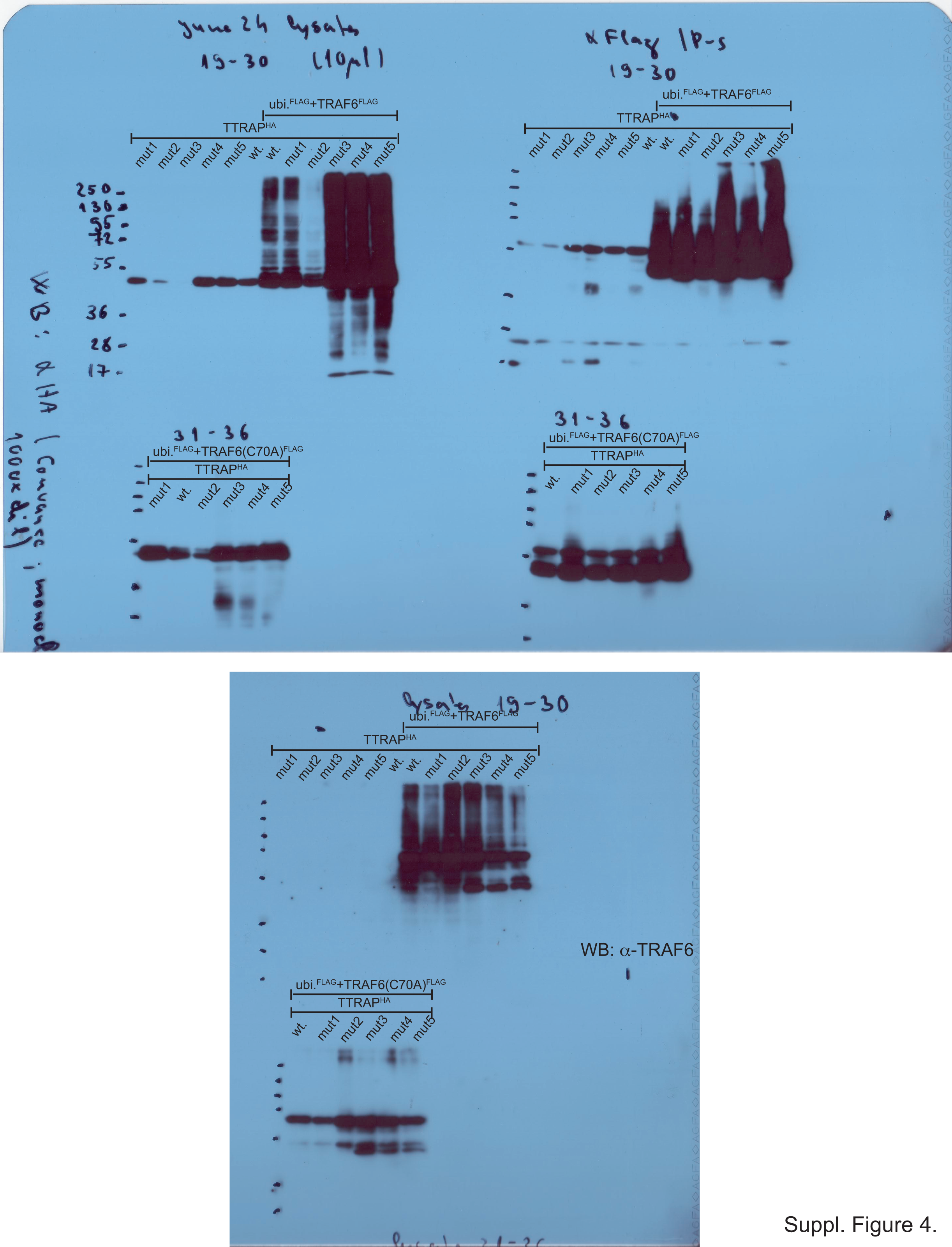

Supplement: Figure S4 — TRAF6 promotes the ubiquitylation of TTRAP. Original scans for Figure 3A. (TIF) [file pone.0025548.s004.tif]
